# Generation of renewable mouse intestinal epithelial cell monolayers and organoids for functional analyses

**DOI:** 10.1186/s12860-018-0165-0

**Published:** 2018-08-15

**Authors:** Emily C. Moorefield, R. Eric Blue, Nancy L. Quinney, Martina Gentzsch, Shengli Ding

**Affiliations:** 1 Department of Cell Biology and Physiology, University of North Carolina at Chapel Hill, 111 Mason Farm Road, 6340C MBRB, CB #7545, Chapel Hill, NC 27599-7545 USA; 2 Marsico Lung Institute/Cystic Fibrosis Research Center, University of North Carolina at Chapel Hill, Chapel Hill, NC USA

**Keywords:** Intestinal epithelial cells, Monolayer, Murine

## Abstract

**Background:**

Conditional reprogramming has enabled the development of long-lived, normal epithelial cell lines from mice and humans by in vitro culture with ROCK inhibitor on a feeder layer. We applied this technology to mouse small intestine to create 2D mouse intestinal epithelial monolayers (IEC monolayers) from genetic mouse models for functional analysis.

**Results:**

IEC monolayers form epithelial colonies that proliferate on a feeder cell layer and are able to maintain their genotype over long-term passage. IEC monolayers form 3D spheroids in matrigel culture and monolayers on transwell inserts making them useful for functional analyses. IEC monolayers derived from the Cystic Fibrosis (CF) mouse model CFTR ∆F508 fail to respond to CFTR activator forskolin in 3D matrigel culture as measured by spheroid swelling and transwell monolayer culture via Ussing chamber electrophysiology. Tumor IEC monolayers generated from the *Apc*^Min/+^ mouse intestinal cancer model grow more quickly than wild-type (WT) IEC monolayers both on feeders and as spheroids in matrigel culture.

**Conclusions:**

These results indicate that generation of IEC monolayers is a useful model system for growing large numbers of genotype-specific mouse intestinal epithelial cells that may be used in functional studies to examine molecular mechanisms of disease and to identify and assess novel therapeutic compounds.

## Background

The goal of intestinal epithelial cell culture is to model intestinal physiology and disease in vitro. Traditional methods that have been used to study intestinal epithelial cell function and intestinal physiology in vitro include immortalized cell lines, 3-dimensional (3D) organoid culture and directed differentiation of pluripotent stem cells (PSC). Immortalized intestinal epithelial cell lines are derived from cancerous tissue or from normal tissue that has been transformed with an oncogene to allow extensive expansion as a monolayer. These lines of cells propagate quickly and some are able to form differentiated, functional intestinal epithelial cell types. For example, the human colorectal adenocarcinoma cell line Caco-2 can spontaneously differentiate into enterocytes and has been successfully used in barrier function assays [1]. However, immortalized intestinal epithelial cell lines demonstrate many limitations including genetic instability and aneuploidy in some lines. Their extensive differentiation time or complete lack of differentiation has resulted in limited usefulness to model intestinal physiology. The development of 3D matrigel culture systems has allowed for the expansion and differentiation of normal intestinal epithelial cells in vitro and is a powerful tool to study intestinal epithelial stem cell (IESC) differentiation and function [2]. Since expansion of organoids from single Lgr5-positive intestinal stem cells (ISCs) was described in Sato et al. [2], 3D organoid culture has been applied in modelling human colorectal cancer [3–5], personalized cancer therapy and drug screening [6, 7] and regenerative medicine [8, 9]. Recent publications have demonstrated the ability of organoids to create a polarized epithelial monolayer that is capable of transport in a transwell culture system, which makes it a useful model for mechanistic studies of barrier function as well as interactions between intestinal epithelial cells and microbes or immune cells [10, 11]. However, 3D organoid culture is technically challenging, requires expensive growth factors, and has the limitation of accessing only the apical side of the cells when assessing interaction with microorganisms or other components [11, 12]. Directed differentiation of PSC sources such as embryonic stem (ES) cells and induced pluripotent stem (iPS) cells can also be used to create functional intestinal epithelium [13]. PSC are grown under conditions that mimic normal in vivo development and undergo step-wise differentiation that result in normal intestinal epithelium useful for studying the molecular basis of intestinal disease. However, these cultures are characterized as fetal in nature, which limits their usefulness in adult functional assays of mature intestinal epithelium [13, 14]. In addition, these cultures are expensive, complex and time-consuming.

Conditional reprogramming has been recently introduced as a method to indefinitely propagate multiple epithelial cell types in vitro by co-culture with irradiated fibroblast feeder cells and the Rho kinase (ROCK) inhibitor Y-27632 [15]. Under these conditions, epithelial cells are induced to an adult stem cell-like state that allows expansion and maintenance of lineage commitment [15, 16]. Success has been demonstrated with a variety of epithelial cell types including prostate, mammary and lung epithelium with limited success in liver and colon epithelium [15]. This exciting advance has allowed expansion and in vitro study of previously difficult to grow cell sources. Recent reports have demonstrated successful cloning and propagation of ground state human intestinal stem cells (ISC^*GS*^) using similar culture conditions including a feeder cell layer and ROCK inhibitor [17]. ISC^*GS*^ differentiated by air liquid interface (ALI) culture and treated with *C. difficile* toxins produced an inflammatory response demonstrating their functional capacity [17]. Cultures that allow the ability to expand intestinal epithelial cells from human biopsies provide a valuable cell source for disease modeling and regenerative medicine.

Long-term expansion of untransformed intestinal epithelium from genetic mouse models as a monolayer would provide a new platform for assays of intestinal physiology and mechanistic studies that have previously been very difficult. Genetic mouse models are available for many disorders affecting the gastrointestinal tract including cystic fibrosis (CF) and intestinal carcinoma. Cystic fibrosis (CF) affects mucus producing epithelium including lung and intestine and is caused by mutations in the cystic fibrosis transmembrane conductance regulator (*CFTR*) gene. The most common cause of CF is a deletion of phenylalanine at position 508 (CFTR ∆F508) that causes protein misfolding and early degradation which prevent CFTR from reaching the plasma membrane and make it nonfunctional [18]. Recent studies have demonstrated the effective use of intestinal organoids derived from primary intestinal biopsy in functional CFTR assays [19]. Mutation in the adenomatous polyposis coli (APC) gene results in the formation of spontaneous intestinal cancers. The *Apc*^Min/+^ mouse model [20, 21], which carries loss of Apc function, causes constant Wnt stimulation that leads to increased expression of β-catenin dependent genes that are associated with cell cycle, resulting in excess intestinal epithelial cell proliferation and adenoma formation in the small intestine and colon [22].

Primary cultures of intestinal epithelium from genetic mouse models achieved by conditional reprogramming provide a physiologically relevant approach to study the mechanisms and novel therapeutics for diseases including CF and intestinal tumorigenesis. Our goal was to achieve long-term culture of untransformed IEC and permit functional studies in vitro. Using a slight modification of the previously reported conditional reprogramming protocol [15], we derived 2D mouse intestinal epithelial monolayers (IEC monolayers) from frozen biopsies of wild-type (WT), CFTR ∆F508 and *Apc*^Min/+^ mouse small intestines. IEC monolayers demonstrated rapid monolayer growth, epithelial phenotype and maintenance of genotype with passage. IEC monolayers derived from these genetic mouse models retain functionality as demonstrated by decreased response of CFTR ∆F508 IEC monolayers to CFTR activation and increased growth rate of *Apc*^Min/+^ IEC monolayers. We conclude that culture under slightly modified conditional reprogramming conditions allows long-term propagation of untransformed, functional monolayers of mouse intestinal epithelial cells from genetic models that may be used in functional studies to examine the physiology of intestinal disorders and to identify effective treatments.

## Methods

### Mice

CFTR ∆F508 mice on C57BL/6 N background were obtained from UNC Cystic Fibrosis Center Mouse Core. *Apc*^Min/+^ mice on C57BL/6 background were originally purchased from The Jackson Laboratory (Bar Harbor, ME), and breeding was continued at the University of North Carolina (Chapel Hill). All animals were maintained in accordance with the Institutional Animal Care and Use Committee (IACUC) (protocol #: 16–193) of the University of North Carolina.

### Mouse tissue harvesting and cryopreservation

After the intestine tissue was dissected, the whole intestine was flushed with ice cold Phosphate buffered saline (PBS) three times. Intestine tissues were cut open longitudinally. Full thickness proximal duodenum (0.5 cm) was isolated from WT or CFTR ∆F508 mice between 6 weeks to 5 months of age and small intestinal tumors were isolated from *Apc*^Min/+^ animals at 4 months of age. Both male and female mice were used. Full thickness small intestine or tumor was minced into pieces less than 3 mm in size using a razor blade. The minced tissue was re-suspended in Freezing Medium (90% fetal bovine serum (FBS) (Gemini, Sacrament, CA)/ 10% DMSO (*v*/v; Sigma-Aldrich, St. Louis, MO)/ 10 μM Y-27632 (Enzo Life Sciences, Farmingdale, NY), frozen in an isopropanol chamber at − 80 °C overnight and transferred to liquid nitrogen for long term storage.

### Tissue processing

Minced, frozen tissue was processed as previously described [15]. Briefly, tissue was thawed at 37 °C and washed by addition of Wash Medium (Dulbecco’s modified Eagle’s medium (DMEM) high glucose containing 10% FBS (Gemini, Sacrament, CA), 100 μg/mL penicillin, 100 μg/mL streptomycin, 100 μg/mL Glutamax, 2.5 μg/ml Amphotericin B (Life Technologies, Carlsbad, CA) and 10 μM Y-27632 (Enzo Life Sciences, Farmingdale, NY) and collected by gravity sedimentation. Minced tissue was then re-suspended in F medium (3:1 (*v*/v) F-12 Nutrient Mixture (Ham) – DMEM high glucose, 5% FBS, 0.4 μg/mL hydrocortisome (Sigma-Aldrich, St. Louis, MO), 5 μg/mL insulin (Sigma-Aldrich, St. Louis, MO), 8.4 ng/mL cholera toxin (Sigma-Aldrich, St. Louis, MO), 10 ng/mL EGF (Life Technologies, Carlsbad, CA), 10 μM Y-27632 (Enzo Life Sciences, Farmingdale, NY)) containing 2.4 mg/mL Collagenase Type I (Sigma-Aldrich, St. Louis, MO) and 0.3 Units/mL Dispase (Corning, Corning, NY) and was incubated at room temperature for 25 min with rocking. Tissue was broken down by vigorous pipetting and filtered through a 70 μm cell strainer (Fisher Scientific, Waltham, CA). Cells were washed with Wash Medium and collected by centrifugation at 1500 rpm for 5 min. Cells were re-suspended in F medium containing 50 ng/ml EGF (R&D Systems, Minneapolis, MN), 100 ng/ml Noggin (Peprotech, Rocky Hill, NJ) and 1 μg/ml R-spondin (R&D Systems, Minneapolis, MN) and plated onto previously prepared Purcol (Advanced Biomatrix, Carlsbad, CA) coated dishes with single layer of irradiated 3T3-J2 feeder cells.

### IEC monolayer maintenance

Approximately 24 h after the initial seeding, F medium containing growth factors (EGF, Noggin, R-spondin) was removed and medium was changed to F medium alone with no added growth factors. IEC monolayers were then maintained long-term in F medium alone with medium changes every other day [5]. IEC monolayers were passaged at confluence approximately every 3–4 days using double trypsinization at a ratio of approximately 1:6 onto a fresh irradiated 3T3-J2 feeder layer in F medium alone.

### Feeder cell preparation

Swiss 3T3 mouse fibroblast (J2 strain) feeder cells [23, 24] were prepared as previously described [15]. Briefly, live 3T3-J2 cells were maintained in a 37 °C incubator with 5% CO_2_ in Complete Medium (DMEM high glucose containing 10% FBS (Gemini, Sacrament, CA), 100 μg/mL penicillin, 100 μg/mL streptomycin, and 100 μg/mL Glutamax (Life Technologies, Carlsbad, CA)). Feeder cells were removed from flasks at 80–90% confluence using 0.05% trypsin-EDTA (Life Technologies, Carlsbad, CA), re-suspended in Complete Medium at a concentration of 5 × 10^6^ cells per ml and irradiated at 30 Gy in an RS-2000 (Rad Source, Suwanee, GA) machine. Irradiated 3T3-J2 cells were plated onto Purcol (Advanced Biomatrix, Carlsbad, CA) coated dishes to achieve 80% confluence and were allowed to attach for at least 4 h before the addition of intestinal epithelial cells.

### Immunofluorescence staining

IEC monolayers were plated under maintenance conditions mentioned above onto glass slides. After 2 days, IEC monolayers on slides were washed 2 times in 1XPBS for 5 min each, fixed in 4% paraformaldehyde for 15 min at room temperature and washed again with 1XPBS for 5 min each. Cells were blocked using 5% normal goat serum for 1 h at room temperature. E-cadherin (1:100; Cat# 3195; Cell Signaling Technology, Danvers, MA) and ZO-1 (1:25; Clone# R40.76; a kind gift from Dr. Alan Fanning; UNC-Capel Hill) antibodies were diluted in permeabilization buffer (5% normal goat serum/0.5% Triton-X in PBS) and incubated on cells overnight at 4 °C. Following antibody incubation, cells were washed 3 times in 1XPBS for 5 min each. Secondary antibodies were diluted in permeabilization buffer (1:300; Cat#111–165-003, Cat# 112–165-003; Jackson Labs, Bar Harbor, ME) and incubated with cells for 2 h at room temperature in the dark. Cells were washed 2 times with 1XPBS for 5 min each. Slides were mounted using DAPI mounting medium (Vector Labs, Burlingame, CA) and imaged using a fluorescent microscope (Olympus IX81; Olympus, Center Valley, PA).

### Flow cytometry

IEC monolayers were separated from 3T3-J2 feeder cells by double trypsinization, washed once with PBS and re-suspended in Staining Buffer (PBS, 1% FBS (Gemini, Sacrament, CA)) at a concentration of approximately 1 × 10^6^ per ml. Cells were incubated with a PE conjugated EpCAM antibody (1:100; clone G8.8; Biolegend, San Diego, CA) for approximately 30 min on ice. Cells were washed twice in Staining Buffer and analyzed using a Cyan flow cytometer and Summit v4.3 software (Beckman Coulter, Brea, CA). EpCAM expression was assessed in IEC monolayers from 3 individual mice. 3T3-J2 feeder cells were used as negative controls.

### Polymerase chain reaction (PCR)

Genomic DNA was extracted from IEC monolayers and 3T3-J2 feeder cells using the DNeasy kit (Qiagen, Valencia, CA) according to the manufacturer’s protocol. Genotyping was performed by combining Apex Taq DNA Polymerase 2× Master Mix (Genesee Scientific, Morrisville, NC) with 0.5 nM of each primer and genomic DNA. *Apc*^Min/+^ primer sequences are: APC F: 5′-TTCTGAGAAAGACAGAAGTTA-3′ and APC R: 5′-TTCCACTTTGGCATAAGGC-3′. The PCR conditions were 95 °C for 15′ followed by 40 cycles of 95 °C for 30″, 59 °C for 30″ and 72 °C for 1′ and an extension of 72 °C for 5′. CFTR ∆F508 genotyping was performed according to the Jackson Labs (Bar Harbor, ME) protocol. CFTR ∆F508 primer sequences are: IMR0269: 5′-TTCAAGCCCAAGCTTTCGCGAG-3′, IMR0270: 5′-CTCCCTTCTTCTAGTCACAACCG-3′ and IMR0271: 5′-CATCTTGATAGAGCCACGGTGC-3′. The PCR conditions were 94 °C for 3′ followed by 12 cycles of 92 °C for 20″, 64 °C for 30″ and 72 °C for 35″ decreasing the annealing temperature by 0.5 °C each cycle. Then 25 cycles of 94 °C for 20″, 58 °C for 30″ and 72 °C for 35″ were completed followed by an extension of 72 °C for 2′.

### Three-dimensional (3D) matrigel culture of IEC monolayers

After double trypsinization, IEC monolayers were counted and approximately 5000 IEC monolayers, which might include small amount of feeder cells, were seeded in 10 μl Low Growth Factor Matrigel (Corning, Corning, NY) and then were loaded in the center of the well of pre-warmed 48 well plate. After polymerization by incubating at 37 °C for 20 min, 350 μl Advanced DMEM-F12 containing N2 supplement without vitamin A, B27 supplement, 10 mM HEPES, 100 μg/mL penicillin, 100 μg/mL streptomycin, 100 μg/mL Glutamax (Life Technologies, Carlsbad, CA), 50 ng/ml EGF (R&D Systems, Minneapolis, MN), 100 ng/ml Noggin (Peprotech, Rocky Hill, NJ) and 1 μg/ml R-spondin (R&D Systems, Minneapolis, MN) was added to each well. Medium was changed every other day with growth factors at the same concentrations as the initial plating except R-spondin, which was reduced to 500 ng/mL.

### Spheroid stimulation assay and swelling quantification

WT and CFTR ∆F508 spheroids were grown in matrigel culture for 4–5 days after seeding, and then treated with forskolin to activate CFTR. A subset of WT spheroids were pretreated for 3 h with 50 μM CFTR_inh_-172 to block CFTR function prior to forskolin treatment. WT and CFTR ΔF508 spheroids received a medium change with or without the addition of 5 μM forskolin. Spheroids were imaged at time 0 and 60 min after the medium change using an inverted fluorescent microscope (IX83; Olympus, Center Valley, PA). Spheroid swelling was quantified by measuring spheroid diameter using Image J software (NIH, Bethesda, MD). The percent increase in spheroid surface area at time 60 min relative to time 0 was calculated. Forskolin response was tested in WT IEC monolayers from 3 individual mice and CFTR ΔF508 IEC monolayers from 3 individual mice.

### Transwell culture conditions

WT and CFTR ∆F508 IEC monolayers were plated in F medium on 12-well Snapwell inserts (Costar, Fisher, Pittsburg, PA) at a concentration of approximately 300,000 cells per insert. Twenty four hours after plating, the medium was changed to Advanced DMEMF-12 containing 20% FBS (Gemini, Sacrament, CA), 100 μg/mL penicillin, 100 μg/mL streptomycin, and 100 μg/mL Glutamax (Life Technologies, Carlsbad, CA) and 10 μM DAPT (Stem Cell Technologies, Vancouver, Canada) to induce differentiation. Transwells were maintained by changing medium every other day and electrophysiological analysis was performed 6–8 days after seeding until cells become confluent.

### Electrophysiological measurements

Transepithelial Cl^−^ secretion in IEC monolayers monolayer cultures were measured in modified Ussing chambers using Acquire & Analyze Software (Physiologic Instruments, San Diego, CA). Chamber temperature was maintained at 36 °C ± 1^o^ C by a circulating water bath, and agar bridges were equilibrated in 5 mL bilateral Krebs bicarbonate ringers solution (KBR; 120 mM Cl^−^, 25 mM HCO^3−^, 140 mM Na^+^, 5.2 mM K^+^, 1.2 mM Ca^2+^, 1.2 mM Mg^2+,^ 2.4 mM HPO_4_^2-,^ 0.4 mM H_2_PO_4_^−^) with 5 mM mannitol (mucosal), 5 mM D-glucose (serosal), and circulated with 95% O_2_ and 5% CO_2_ gas, at pH = 7.4. Prior to data acquisition, IEC monolayers cultures were mounted and reference measurements for potential difference (PD) and transepithelial resistance (Rt) were recorded. IEC monolayers cultures were voltage clamped, and measurements were recorded digitally every 20 s. Cultures were equilibrated for a minimum of 10 min prior to the addition of agonists or inhibitors. Compounds (obtained from Sigma, except UTP from GE Healthcare) were added in order as indicated: amiloride, 100 μM (mucosal) to inhibit ENaC; forskolin, 10 μM/ IBMX, 100 μM (mucosal and serosal) to induce cAMP activation of CFTR, UTP 100 μM (mucosal) to activate Ca^2+^-activated Cl^−^ channels (CaCC), and GlyH-101 (50 μM, mucosal) to inhibit Cl^−^ channel current. Data was imported and processed in Microsoft Excel and Origin 9.0.0. (OriginLab Corporation). Electrophysiological measurements were performed on IEC monolayers derived from 2 individual WT mice and 3 individual CFTR ∆F508 mice.

### Quantification of IEC monolayers colony expansion

WT and *Apc*^Min/+^ Tumor IEC monolayers were plated onto feeder-coated dishes in F medium. Cells were maintained by changing F medium every other day. On day 2 after plating, IEC monolayers colonies were identified and imaged using an inverted fluorescent microscope with an incubation chamber (IX83; Olympus, Center Valley, PA). The same IEC monolayers colonies were tracked and imaged at days 3 after plating. IEC monolayers colony size was quantified at each time point by measuring colony diameter using ImageJ software (NIH, Bethesda, MD). Colony expansion was assessed in 3 individual WT IEC monolayers and 3 individual *Apc*^Min/+^ tumor IEC monolayers.

### Quantification of IEC monolayers spheroid growth

WT and *Apc*^Min/+^ tumor IEC monolayers were passaged into matrigel (Matrigel Matrix, Corning, Cat# 354230, New York) to grow as spheroids, where they were tracked and measured to assess growth rate. At day 3, 5 and 7 after plating, images from the spheroids were taken from WT and *Apc*^Min/+^ tumor IEC monolayers spheroids using an inverted fluorescent microscope (IX85; Olympus, Center Valley, PA). Spheroid size was quantified at each time point by measuring spheroid diameter using ImageJ software (NIH, Bethesda, MD). Spheroid growth was assessed in IEC monolayers derived from 3 individual WT and 3 individual *Apc*^Min/+^ tumor samples.

### Statistical analysis

Data represent means ± SEM. Data was analyzed using Student’s t-test in Graphpad Prism 5.0. *P <* 0.05 was considered statistically significant.

## Results

### Long-term expansion of normal adult mouse small intestinal epithelium culture under modified conditional reprogramming conditions

**Fig. 1:**
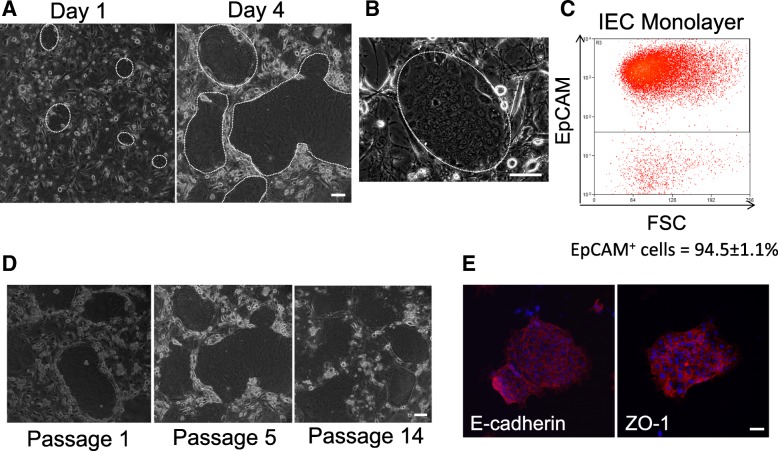
Mouse small intestinal epithelial cells divide and maintain morphology with passage and express epithelial cell markers when cultured under modified conditional reprogramming conditions. **a** Representative phase contrast images of IEC monolayer colonies 1 day post-plating and 4 days after post-plating (passage 12). Dashed circle indicates colonies. Images were taken at 10× magnification. Scale bar: 100 μm. **b** Representative image of colony with higher magnification (20×) **c** Representative flow cytometry plot of EpCAM expression in IEC monolayers. Data represents IEC monolayers from 3 individual mice and diverse passages. **d** Representative phase contrast images of IEC monolayers colonies at early, middle and later passages. **e** Representative fluorescence microscopy images of IEC monolayers stained with tight junction markers E-cadherin and ZO-1

Based on previous successful reports of conditionally reprogramming variety of epithelial cell types [15] and selective expansion of human ground state intestinal stem cells (ISC^*GS*^) [17], we aimed to expand mouse intestinal epithelium using similar conditions. We applied modified conditional reprogramming culture conditions to mouse small intestine or small intestinal tumor to achieve long-term growth of intestinal epithelial monolayers. Culture of cryopreserved mouse intestine with ROCK inhibitor and irradiated 3T3-J2 feeder cells with initial addition of growth factors critical in intestinal development and intestinal stem cell maintenance resulted in mouse intestinal epithelial monolayers (IEC monolayers) having consistent cuboidal morphology and growing in tight, rapidly expanding colonies in early, and later passages (Fig. 1a, b, d). To date, IEC monolayers have shown the ability to grow under modified conditional reprogramming conditions for up to 3 months with no change in morphology or passaging rate. The epithelial nature of IEC monolayers was confirmed by using flow cytometry to test for the presence of the epithelial specific surface marker EpCAM. Around 95% of collected IEC monolayers expressed EpCAM demonstrating that we are able to specifically expand epithelial cells from the mouse small intestine (Fig. 1c). The remainder of the cells not expressing EpCAM (approximately 5% of the culture) may be residual feeder cells which are mesenchymal in origin. Formation of tight junctions was confirmed by immunofluorescent staining for E-cadherin and ZO-1 (Fig. 1e).

### Maintenance of mutant allele

**Fig. 2:**
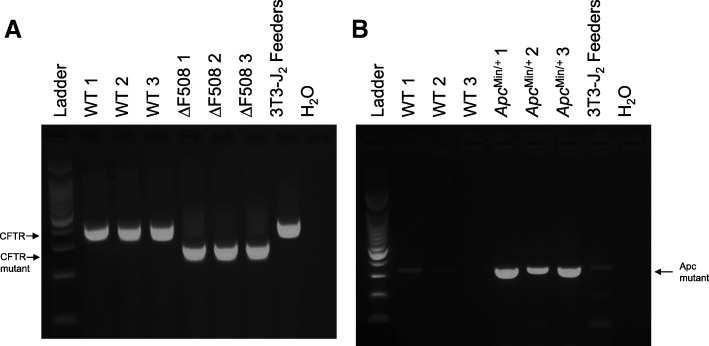
Small intestinal IEC monolayers from WT, CFTR ∆F508 and *Apc*^Min/+^ mice maintain presence of mutant allele across passages. **a** Genomic DNA isolated from IEC monolayers derived from wild-type (WT) or cystic fibrosis (CFTR ∆F508) mouse models amplified by CFTR primers. WT IEC monolayers and irradiated 3T3-J2 feeders contain the normal length CFTR gene and CFTR ∆F508 IEC monolayers contains the truncated CFTR gene. **b** Genomic DNA isolated from IEC monolayers derived from WT or intestinal cancer (*Apc*^Min/+^) mouse models. IEC monolayers from *Apc*^Min/+^ mouse model contains the mutant Apc gene. *n* = 3 individual animals in each genetic mouse model

Maintaining presence of a parental mutant allele across passage is critical for the usefulness of IEC monolayers in functional assays. Genomic DNA was isolated from IEC monolayers following several passages to determine the ability of IEC monolayers to retain the mutation present in the parental intestinal epithelium across passage. Figure 2a indicates that WT IEC monolayers maintained the presence of the normal CFTR gene and IEC monolayers derived from CFTR ΔF508 animals maintained the CFTR ∆F508 mutation over prolonged passaging (passage 11–13). In the model of intestinal cancer, we observed maintenance of the mutated APC gene in IEC monolayers derived from intestinal tumors of *Apc*^Min/+^ animals (Fig. 2b).

### Functional studies of CFTR ΔF508 mouse IEC monolayers

**Fig. 3:**
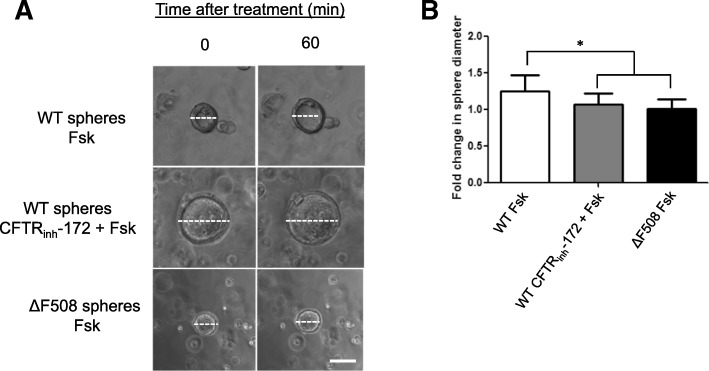
Mouse CFTR ∆F508 IEC monolayers retains CFTR dysfunction in intestinal epithelium. **a** Representative images of WT and CFTR ∆F508 IEC monolayer derived spheroids grown in matrigel and treated with forskolin (Fsk) to induce spheroid swelling. Images were taken at 10× magnification. Scale bar: 100 μm. White dashed line: representation of spheroid diameter measurement. **b** Quantification of WT and CFTR ∆F508 IEC monolayers swelling in response to CFTR activation (Forskolin, Fsk) and inhibition (CFTR_inh_-172). *p < 0.05, *n* = 3 individual animals in each treatment group

**Fig. 4:**
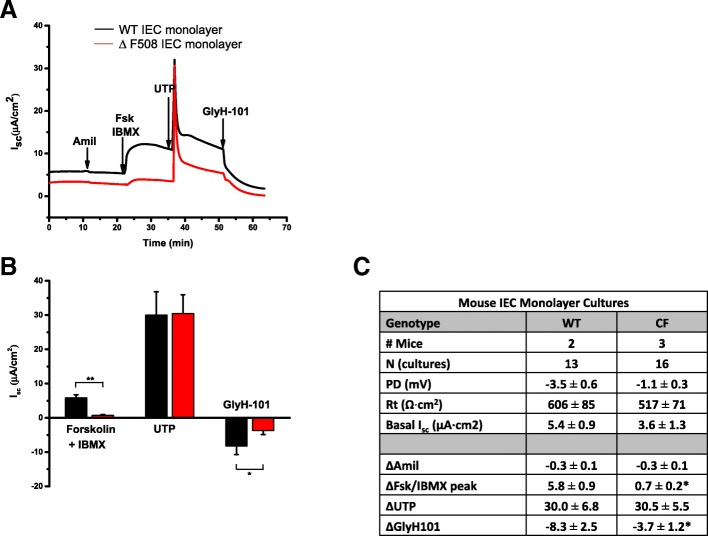
Electrophysiological measurements of WT and CF mouse IEC monolayers. **a** Representative traces of electrophysiological measurements of WT and CF mouse IEC monolayers in Ussing chambers. Amiloride (Amil), Forskolin + IBMX (Fsk/IBMX), UTP and GlyH-101 were added sequentially. **b** Mean Forskolin/IBMX, UTP and GlyH101 responses measured in Ussing chambers. Electrophysiological measurements were performed on IEC monolayers derived from 2 individual WT mice and 3 individual ∆F508 CFTR mice. **c** Measured bioelectric characteristics of WT and CF IEC monolayer mouse small intestinal cultures. Basal potential difference (mV), transepithelial resistance (Ω x cm^2^), and current (μA/cm^2^) are reported. Data represented at mean ± SEM based on cultures. *p < 0.05

IEC monolayers derived from WT and CFTR ∆F508 animals were used to assess CFTR functionality in long-term intestinal epithelial cell culture. IEC monolayers were expanded as monolayers on feeder cells with ROCK inhibitor and then was plated into matrigel and Transwell culture systems to assess their response to CFTR activation. To demonstrate CFTR function, we performed the previously described forskolin-induced swelling assay on WT and CFTR ∆F508 IEC monolayers spheroids grown in matrigel [19]. Forskolin functions by raising the levels of intracellular cAMP which activates CFTR gene and causes fluid secretion into the lumen resulting in swelling of 3D intestinal structures [19]. We observed that IEC monolayers collected from feeder cultures were successfully grown in matrigel, and formed 3D spheroids making them ideal for use in this assay (Fig. 3a). WT IEC monolayer derived spheroids treated with forskolin swelled significantly over the 60 min time period compared to their size prior to treatment revealing functional CFTR (Fig. 3a, b). To demonstrate that forskolin-induced swelling was dependent on CFTR, we pre-treated WT IEC monolayer derived spheroids with the CFTR inhibitor CFTR_inh_-172 then added forskolin and assessed swelling response. WT IEC monolayer derived spheroids with CFTR inhibitor treatment do not swell significantly in the presence of forskolin indicating that this response is CFTR dependent (Fig. 3a, b). CFTR ∆F508 IEC monolayer derived spheroids treated with forskolin revealed no significant increase in organoid diameter at 60 min after the addition of forskolin compared to time 0 (Fig. 3a, b), demonstrating the maintenance of dysfunctional CFTR in IEC monolayers derived from CFTR ∆F508 mice. Culture of intestinal epithelial cell lines on microporous membranes such as Transwell inserts has been shown to lead to cell differentiation and polarization [25]. Recent studies have demonstrated the ability of intestinal organoids cultured on Transwells in the presence of γ-secretase inhibitor DAPT to form functional monolayers capable of transport [10]. We densely plated IEC monolayer cells on Transwell inserts with DAPT treatment to achieve a functional intestinal epithelial monolayer. Both WT and CFTR ∆F508 IEC monolayers formed confluent, planar cultures under these conditions allowing electrophysiological assays to test cellular responses to CFTR activating drugs in Ussing chambers. We detected noticeable responses in CFTR-mediated short-circuit current (Isc) to cAMP increase by forskolin and the cAMP phosphodiesterase inhibitor 3-isobutyl-1-methylxanthine (IBMX) in WT but not CF IEC monolayer derived transwell cultures (Fig. 4b, **p < 0.001). We found no statistical difference between IEC monolayer derived transwell cultures from CF vs. WT mice in the amplitude of responses of transient calcium activated Cl- channels (CaCCs) to UTP. Inhibition with GlyH-101, which is known to inhibit CFTR and CaCC activity [26], showed a significant difference in ΔIsc of WT and CF cultures (Fig. 4b *p < 0.05). Figure 4a shows a sample trace from a WT IEC monolayer derived transwell culture (black) and a CFTR ΔF508 IEC monolayers monolayer (red). These observations indicate dysfunctional CFTR from CFTR ΔF508 IEC monolayers (Fig. 4c).

### Functional studies of Apc^Min/+^ mouse tumor IEC monolayers

**Fig. 5:**
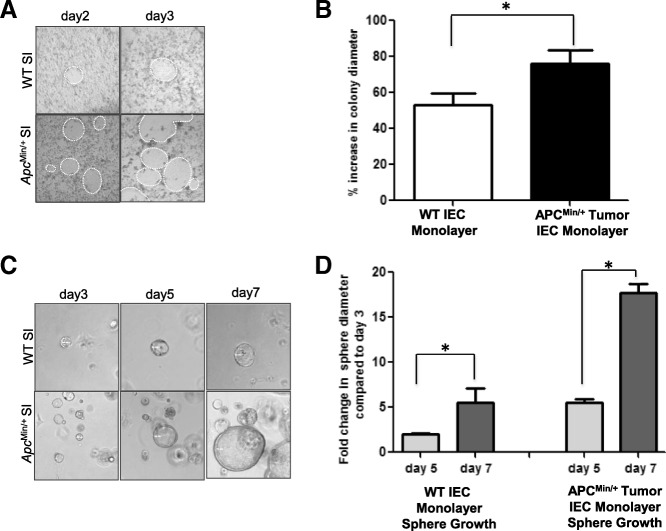
Mouse *Apc*^Min/+^ tumor IEC monolayers retain functional characteristics of intestinal epithelium. **a** Representative images of WT and *Apc*^Min/+^ tumor IEC monolayers colonies growing on a feeder layer at passage 0. **b** Quantification of WT and *Apc*^Min/+^ tumor IEC monolayer colony growth between days 2 and 3 post-plating. **c** Representative images of WT and *Apc*^Min/+^ tumor IEC monolayer derived spheroids grown in matrigel. **d** Quantification of WT and *Apc*^Min/+^ tumor IEC monolayer derived spheroid growth based on spheroid diameter on day 5 and day 7 compared to day 3. *p < 0.05, *n* = 3 individual animals in each genetic mouse model

WT and *Apc*^Min/+^ tumor IEC monolayer growth was evaluated both as a monolayer on 3T3-J2 feeder cells and in the 3-D matrigel system, and then their functionality was assessed. Prior to the first passage (on passage 0 after freshly isolated cell plating), we evaluated the expansion rate of IEC monolayers by measuring the diameter of the growing IEC monolayer colonies over time. *Apc*^Min/+^ tumor IEC monolayers grown as a monolayer under conditional reprogramming conditions demonstrated a faster growth rate as measured by the significantly higher increase in colony diameter between days 2 and 3 compared to WT IEC monolayers (Fig. 5a, b). We also passaged both WT and *Apc*^Min/+^ tumor IEC monolayers into matrigel conditions and assessed the growth rate of WT and *Apc*^Min/+^ IEC monolayer derived spheroids grown in matrigel by measuring spheroid diameter. *Apc*^Min/+^ IEC monolayer derived spheroids grew faster than WT IEC monolayer derived spheroids with significantly higher fold change in spheroid diameter at day 5 and day 7 compared to WT IEC monolayer derived spheroids (Fig. 5c, d).

## Discussion

Here we demonstrate a system to rapidly expand functional intestinal epithelial cells; a process that allows long-term propagation of intestinal epithelial cells in a monolayer format. Conditional reprogramming employs the use of irradiated feeder cells and a Rho kinase (ROCK) inhibitor to induce normal and tumor epithelial cells to long-term proliferation in vitro without the need for exogenous gene transduction [15]. When cultured with ROCK inhibitor Y-27632 in the presence of a fibroblast feeder layer, mouse intestinal epithelial monolayers (IEC monolayers) grow in a monolayer format that is capable of rapid expansion, maintains specific mutant allele and is suitable for functional analyses. IEC monolayers circumvent many of the issues present in current methods of in vitro intestinal epithelial cell culture and provide a way to generate large numbers of untransformed primary intestinal epithelium from a variety of genetic mouse models.

Expansion of IEC monolayers on feeder cells is critical to achieve large cell numbers, but their ability to survive and grow after transfer to additional culture systems is critical for functional analysis of disease in vitro. Primary prostate and mammary cells are rapidly reprogrammed toward an adult stem cell-like phenotype that are able to form prostaspheroids and mammospheroids in matrigel [15]. IEC monolayers are able to form 3D spheroids when transferred to Matrigel culture that are similar in appearance to intestinal epithelial stem cell or crypt derived enterospheres. IEC monolayer derived spheroids have proven valuable in testing CFTR modulator compounds and cell proliferation rates and may also be useful for examining host-pathogen interactions in vitro. When transferred to a semi-permeable insert in a Transwell format, IEC monolayer cells form a confluent monolayer. Culture in the Transwell system allows IEC monolayers to be tested in electrophysiology assays and may be used to study intestinal permeability and transport. Importantly, both the Matrigel and Transwell systems achieve IEC monolayer culture in the absence of a feeder layer which allows for cleaner studies of cell signaling and facilitates more advanced functional studies.

We established IEC monolayers from mice homozygous for the most common CFTR mutation, deletion of phenylalanine at position 508 (∆F508), which causes loss of CFTR function through misfolding, endoplasmic reticulum (ER) retention and early degradation [27]. Mice carrying the ΔF508 mutation display a relatively severe intestinal pathology consistent with the CF related intestinal dysfunction observed in humans [28]. The ∆F508 mutant mouse model has been used to study CFTR processing and transportation defects and to test possible therapeutic compounds [29]. In the current study, we have successfully grown 3D spheroids from WT IEC monolayers and ΔF508 IEC monolayers plated in matrigel conditions. The ability to form sphereoids is a critical element of assessing CFTR functionality. The recently developed forskolin swelling assay creates a platform to directly examine CFTR function in early organoid cultures [19, 30].

Transwell culture is critical for electrophysiological analysis of CFTR function. This is the first time, to our knowledge, that electrophysiology has been successfully preformed on primary monolayer cultures of intestinal epithelial cells. We detected two mechanisms of Cl^−^ secretion: 1) cAMP-mediated by CFTR and 2) purinergic activation of CaCCs. We found a substantial difference in the amplitude of response to forskolin and IBMX, showing that CFTR is present in the small intestine of WT mice and is responsible for a portion of Cl^−^ and/or bicarbonate transport in this region. In both ∆F508 CFTR and WT mice, we found no statistical difference in the amplitude of the Ca^2+^ activated Cl^−^ channel response to the P_2_Y receptor agonist, UTP. The difference in inhibition of short-circuit current by GlyH-101 in WT and CF cultures reveals further evidence that Cl^−^ channels are active in IEC monolayer cultures, but abrogated in CF IEC monolayer cultures.

In previous studies, conditionally reprogrammed cells (CRC) have been created from carcinomas including breast tumors of transgenic mice and DMBA-treated rats allowing for molecular analysis [15]. In clinic, CRC have also been created from tumor epithelium of a recurrent respiratory papillomatosis (RPP) and used to successfully identify an effective treatment [31]. We derived IEC monolayers from the intestinal tumor tissue of *Apc*^*Min/*+^ mouse intestinal cancer model. *Apc*^*Min/*+^ mouse model carries a heterozygous germ line mutation at codon 850 of the tumor suppressor adenomatous polyposis coli (APC) gene [32] and loss of the normal Apc allele in intestinal epithelium leads to intestinal adenoma formation [33]. The *Apc*^Min/+^ mouse model is commonly used to investigate the molecular mechanisms behind initiation and progression of intestinal tumorigenesis and to study preventative and therapeutic approaches to intestinal cancer in vivo as mice develop many adenomas in the small intestine and colon in the first 6 months of life [34]. In the present study, we demonstrate that *Apc*^Min/+^ tumor IEC monolayers retain cancerous phenotype and expand much more quickly under normal growth conditions and in 3D matrigel conditions than WT IEC monolayers. To date, in vitro studies rely on transformed cell lines that are genetically unstable and do not effectively represent tumor physiology [35]. *Apc*^Min/+^ IEC monolayers could be used as an in vitro model to identify the molecular basis of tumor progression and to study factors that may promote or prevent tumor progression. Specifically, *Apc*^Min/+^ IEC monolayers may be used to investigate signaling pathways involved in tumorigenesis by overexpression or knockdown of key genes. Established IEC monolayers are an invaluable tool to study the role of therapeutic substances able to prevent molecular and phenotypic progression of carcinoma.

The goal of our study is to expand normal intestinal epithelium as IEC monolayers and to use these cells in functional assays. However, additional experiments are required to fully characterize IEC monolayers. CRC have been shown to share properties with adult stem cells and keratinocyte CRC express markers consistent with epithelial stem cells [16]. Studies directly comparing the gene and protein expression profiles of IEC monolayers with IESC and intestinal progenitor cells would be useful to determine their characteristics. CRC have also been shown to maintain tissue-specific differentiation potential. Under air-liquid interface (ALI) culture conditions, cervical CRC are able to form a stratified epithelium that expresses proteins of the mature cervix while tracheal CRC differentiate to form a mucociliary airway epithelium [16]. Additional experiments on IEC monolayer derived spheroids and Transwell culture are necessary to determine the full extent of their differentiation.

Taken together, our results support the use of IEC monolayers from genetic mouse models as an in vitro system to study molecular mechanisms causing intestinal disease and to identify effective therapeutics. We have demonstrated the ability of IEC monolayers to be useful in examining CFTR and Apc dysfunction. This system may be expanded to allow the growth of intestinal epithelium from mice with many different genetic mutations to allow physiologically relevant disease modeling. The ability to rapidly expand intestinal epithelium from mutant mouse models will provide a large number of functional cells for a variety of downstream analyses including barrier function, host-microbe interaction and epithelial-stromal interaction. High cell yields from a single biopsy also provide the possibility for longitudinal studies and high throughput screening assays.

## Conclusion

Mutant mouse IEC monolayers constitute an in vitro model to promote understanding of the molecular basis of genetic disease and contribute to the identification of novel therapeutic approaches.

## Abbreviations

ALI: Air liquid interface
APC: Adenomatous polyposis coli
CaCC: Ca^2+^-activated Cl^−^ channels
cAMP: Cyclic adenosine monophosphate
CF: Cystic fibrosis
CFTR: Cystic fibrosis transmembrane conductance regulator
DMEM: Dulbecco’s modified Eagle’s medium
ES: Embryonic stem
FBS: Fetal bovine serum
IACUC: Institutional Animal Care and Use Committee
IEC monolayers: Mouse intestinal epithelial cell monolayers
IESC: Intestinal epithelial stem cell
iPS: Induced pluripotent stem
PBS: Phosphate buffered saline
PD: Difference (PD)
PSC: Pluripotent stem cells
ROCK: Rho kinase
Rt: Transepithelial resistance
WT: Wild-type
